# Municipal green waste as substrate for the microbial production of platform chemicals

**DOI:** 10.1186/s40643-023-00663-2

**Published:** 2023-07-22

**Authors:** Marianne Volkmar, Anna-Lena Maus, Martin Weisbrodt, Jonathan Bohlender, Alexander Langsdorf, Dirk Holtmann, Roland Ulber

**Affiliations:** 1 Institute of Bioprocess Engineering, University of Kaiserslautern-Landau, Gottlieb-Daimler-Straße 49, 67663 Kaiserslautern, Germany; 2 Institute of Bioprocess Engineering and Pharmaceutical Technology, University of Applied Sciences Mittelhessen, Wiesenstraße 14, 35390 Giessen, Germany; 3 Karlsruhe Institute of Technology, Institute of Process Engineering in Life Sciences, Kaiserstraße 12, 76131 Karlsruhe, Germany

**Keywords:** Circular bioeconomy, Biomass valorization, Lignocellulose, Biobutanol, Green waste

## Abstract

In Germany alone, more than 5·10^6^ tons of municipal green waste is produced each year. So far, this material is not used in an economically worthwhile way. In this work, grass clippings and tree pruning as examples of municipal green waste were utilized as feedstock for the microbial production of platform chemicals. A pretreatment procedure depending on the moisture and lignin content of the biomass was developed. The suitability of grass press juice and enzymatic hydrolysate of lignocellulosic biomass pretreated with an organosolv process as fermentation medium or medium supplement for the cultivation of *Saccharomyces cerevisiae*, *Lactobacillus delbrueckii* subsp. *lactis*, *Ustilago maydis*, and *Clostridium acetobutylicum* was demonstrated. Product concentrations of 9.4 g_ethanol_ L^−1^, 16.9 g_lactic acid_ L^−1^, 20.0 g_itaconic acid_ L^−1^, and 15.5 g_solvents_ L^−1^ were achieved in the different processes. Yields were in the same range as or higher than those of reference processes grown in established standard media. By reducing the waste arising in cities and using municipal green waste as feedstock to produce platform chemicals, this work contributes to the UN sustainability goals and supports the transition toward a circular bioeconomy.

**Graphical Abstract:**

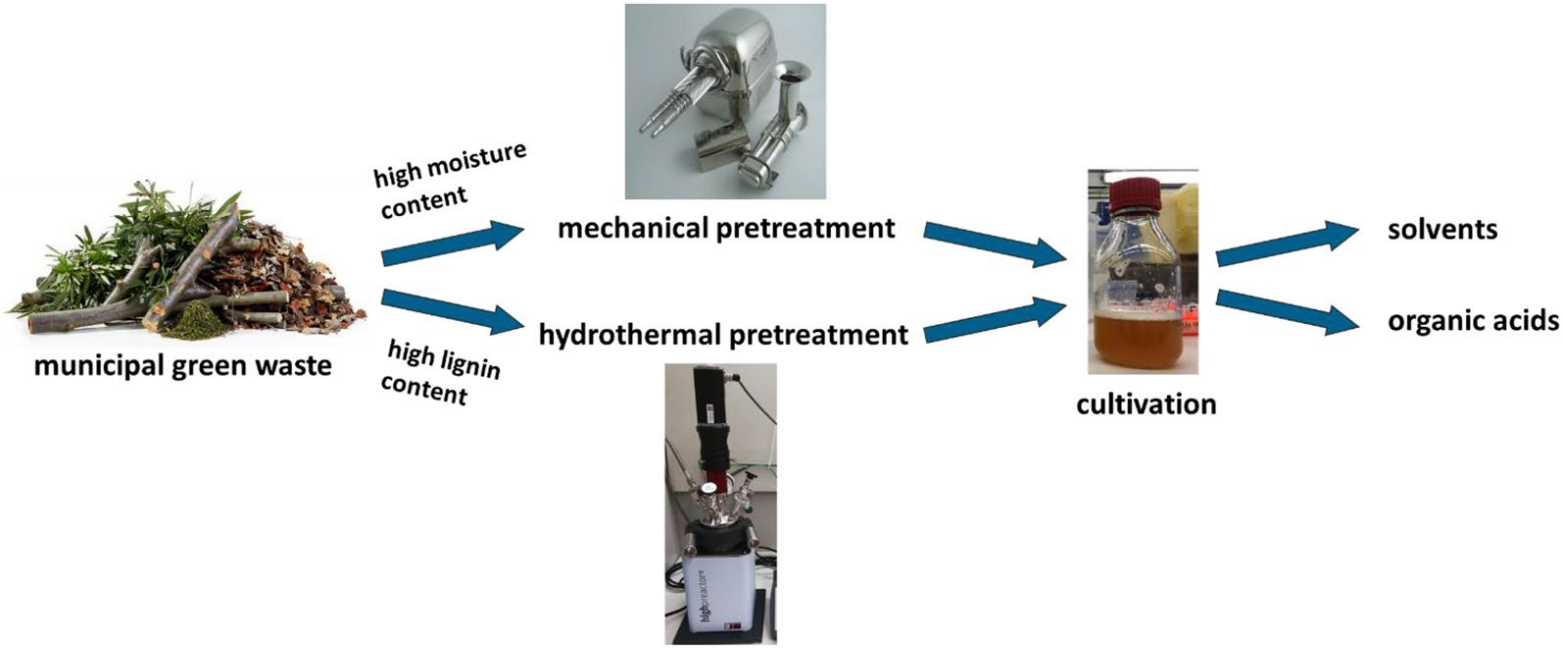

## Introduction

Although frequently used in the literature, there is no standardized definition of municipal green waste. In general, green waste includes all compostable organic fractions of generated waste, including bio-waste from food processing. In this study, the term municipal green waste includes all lignocellulosic biomass generated in urban municipal areas, while excluding food waste. Examples of this municipal green waste are grass clippings, hedge pruning, and tree cuttings including bark as well as leaves (Borisova et al. 2022). This material occurs in municipal areas, for example, in public gardens and parks, cemeteries, and as roadside greenery (Eades et al. 2020). In the European waste catalog, it is summarized under the waste code 200201 as “biodegradable garden and park wastes, including cemetery waste” (Commission decision 2014). To prevent negative effects on the global environment, the EU council directive obliges all member states to reduce the amount of biodegradable waste in landfills (Official Journal of the European Communities 1999; Borisova et al. 2022). According to the classification, 5.7 · 10^6^ tons of municipal green waste were collected in Germany in 2020 (German Federal Statistical Office Destatis 2020). The depletion of fossil fuels combined with the increasing demand for energy and food emphasizes the need for alternative feedstocks. Following the ideal of a circular economy, this inefficiently used waste stream of municipal green waste constitutes a promising resource with so far untapped potential. According to Medick et al. (2017), this resource could be much more productive than the current waste management report indicates. They determined the technical biomass potential of the city of Berlin, Germany, to be more than 120,000 tons of herbaceous fresh matter per year. This includes leaves and grass cuttings which could technically be harvested. So far, the collected biomass is mainly composted or used for the production of biogas. However, this application has not yet been economically viable (Inghels et al. 2016; Pick et al. 2012). In a previous review, alternative valorization methods for municipal green waste were already discussed (Langsdorf et al. 2021).

On the molecular level, the main component of green waste is lignocellulose, a complex structure of cellulose, hemicellulose, and lignin. Cellulose consists of glucose, while hemicellulose is a heteropolymer of hexoses and pentoses. Lignin comprises different phenolic compounds. Additionally, green waste contains proteins, lipids, and minerals (Mohapatra et al. 2017). The exact composition varies greatly. One important factor is the type of biomass. Woodier biomass has a higher lignin content, while herbaceous biomass usually has a lignin content below 25% (Langsdorf et al. 2021). Besides the species, the season and the condition of the soil on which it is grown also influence the composition of the biomass. With up to 60%, carbohydrates are the dominating fraction of lignocellulosic biomass (Álvarez et al. 2016). Therefore, green waste is a promising growth substrate for microorganisms.

Every valorization of municipal green waste requires a pretreatment of the raw material. Especially the recalcitrance of lignin is often challenging. Established procedures employ physical, chemical, or biological methods as well as combinations. Recently, a lot of publications extensively reviewed different pretreatments of biomass with their advantages and disadvantages (Broda et al. 2022; Banu et al. 2021; Martín et al. 2022; Gallego-García et al. 2023; Sołowski et al. 2020; Duque et al. 2021).

There is a long tradition of using lignocellulosic biomass as feedstock. To bypass the food or feed dilemma, most processes use agricultural waste streams such as straw. As described above, municipal green waste is not only lignocellulosic biomass and arises in large amounts, but also has no other application so far besides the unprofitable biogas production and is therefore in no competition with other usages.

In literature, there are various reports of using lignocellulosic biomass as feedstock for fermentations. Most of these relate to agricultural waste streams, but there are also publications dealing with material that can be compared to municipal green waste. For example, Schoeters et al. used press juice from a mixture of *Festulolium* and perennial ryegrass for the co-cultivation of microalgae to produce high-protein biomass (Schoeters et al. 2023). Langsdorf et al. prepared growth media from grass clippings either by using a juice extractor or by blending the feedstock and produced α-humulene with *Cupriavidus necator* without any further additives (Langsdorf et al. 2022). The press juices of *Miscanthus x giganteus* (MxG), *Lolium perenne*, and grass-clover mixtures were also already used for cultivations (Boakye-Boaten et al. 2016; Hull et al. 2014; Santamaria‐Fernandez et al. 2019).

The aim of this study is to examine the potential of municipal green waste as a feedstock for microbial production of different platform chemicals. Therefore, suitable methods for the pretreatment of lignocellulosic biomass were selected and a process scheme for the usage of municipal green waste was developed. The resulting components were analyzed and used as solely cultivation media or as media supplements. To cover various fermentation requirements, the cultivation of *Saccharomyces cerevisiae*, *Lactobacillus delbrueckii* subsp. *lactis*, *Ustilago maydis*, and *Clostridium acetobutylicum* was tested for the production of platform chemicals such as the solvents acetone, butanol, and ethanol as well as lactic acid and itaconic acid. Thus, this work contributes to the UN sustainability development goals (SDG), especially to provide affordable and clean energy (SDG 7) as well as to develop sustainable cities and communities (SDG 11).

## Results and discussion

### From parks to biorefineries

In municipal areas, green waste arises mainly in public gardens and parks as well as through roadside greenery. Collected by the city authorities, it can be directly used for a biorefinery application without further pre-processing. However, it might be necessary to remove urban contaminations (e.g. plastics, printed paper) or stones which occur in municipal parks.

### Pretreatment of municipal green waste for the use in biorefineries

**Fig. 1:**
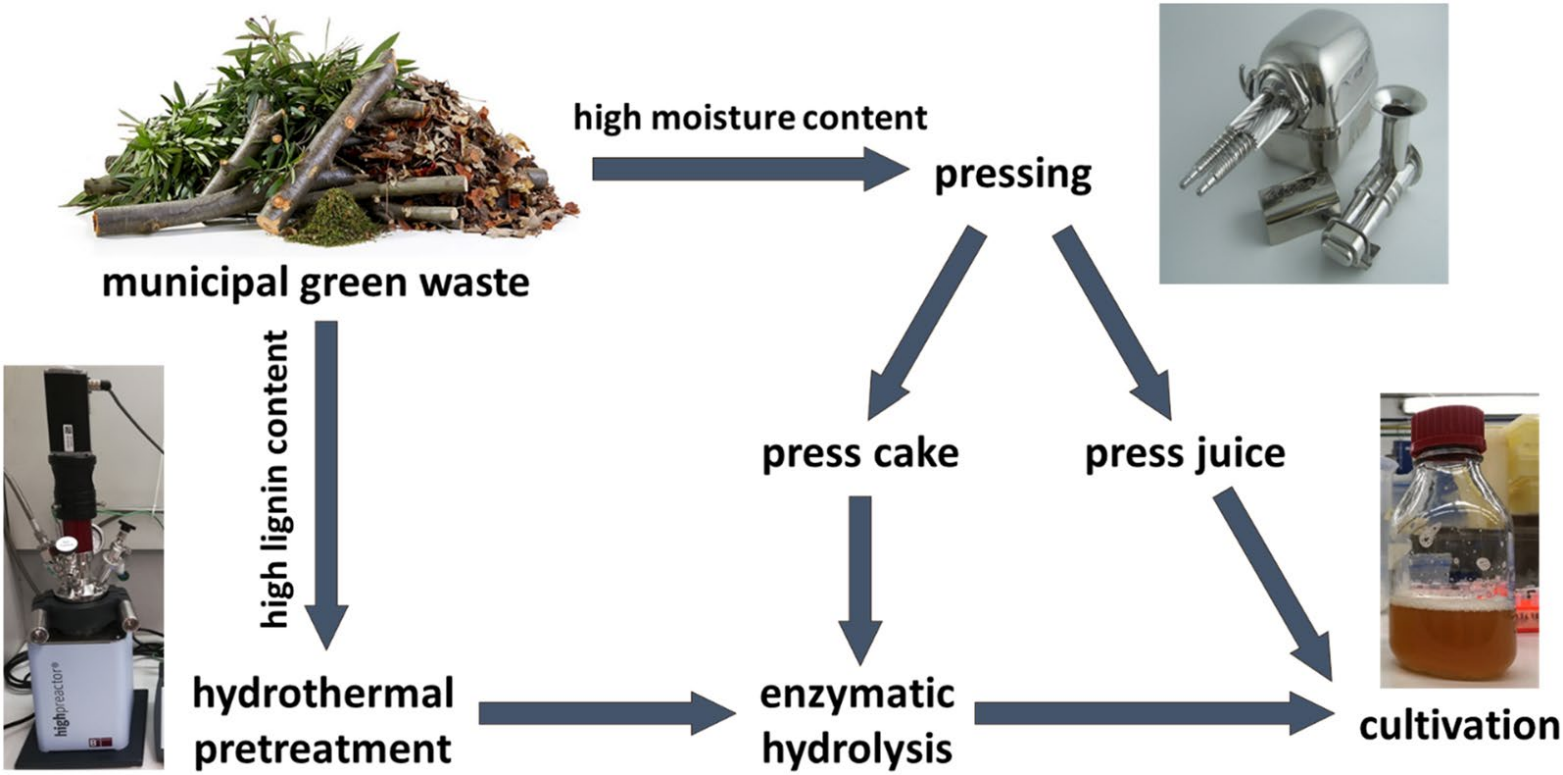
Process schematic of different pretreatment methods of municipal green waste. Biomass with a high lignin content is pretreated using an organosolv process to break up the recalcitrant lignocellulose structure. The residue is saccharified enzymatically before the use in cultivations. Biomass with a high moisture content is separated into a liquid press juice and a solid press cake. The press juice can directly be used for cultivations; the press cake undergoes enzymatic saccharification together with the residue of the high-lignin biomass pathway after organosolv pretreatment

A suitable pretreatment of the biomass is necessary to exploit the carbohydrates and nutrients comprised in municipal green waste for further use in a biorefinery. A process schematic for the pretreatment of municipal green waste according to the different properties of the lignocellulosic material for the use in microbial cultivations was established. The different pathways are summarized in Fig. 1. The most important factor for the pretreatment is the moisture content of the raw material. Feedstock with a dry matter content of less than 40% can be pressed. In this way, the biomass is separated into two different fractions, namely, a solid press cake, and a liquid press juice. The latter can directly be used as a medium or medium supplement for microbial cultivations. Due to its low moisture content, the press cake is easier to store and transport than the initial biomass. This simplifies the logistics behind a biorefinery concept that uses municipal green waste and makes its implementation more realistic. A dry matter share of more than 40% is usually accompanied by an elevated lignin content. For example, wood cuttings of the hardwood beech contain 26% lignin (Smit et al. 2022), and the softwood pine 37% lignin (Kundu et al. 2021), while the lignin content of herbaceous biomass with a lower dry matter share is typically below 25% (Langsdorf et al. 2021).

If the pressing step of biomass with a high moisture content includes a harsh mechanical pretreatment, as is the case with for example a screw press system, an additional hydrothermal process step is not necessary (Varriale et al. 2022). The solid residue of the pressed biomass, the so-called press cake, can be saccharified enzymatically, without further processing, and together with the residue of the low-moisture pathway. Biomass with a high lignin content needs to undergo hydrothermal pretreatment. Hereby, the recalcitrant structure of lignocellulose is broken up through the application of elevated temperature and pressure. As lignin is more soluble in organic solvents (Jääskeläinen et al. 2017), these are used during hydrothermal pretreatments to effectively remove lignin from the remaining biomass in the so-called organosolv processes (Paulsen Thoresen et al. 2023; Parot et al. 2022). Varriale et al. showed a 41.1% increase in carbohydrate yield from enzymatic hydrolysis of mixed wood chips after an organosolv treatment compared to untreated material (Varriale et al. 2022). The solid residue of hydrothermal procedures is washed to remove by-products such as degraded carbohydrates.

To obtain sugar monomers, the residue is saccharified enzymatically. In this enzymatic hydrolysis, saccharolytic enzymes such as cellulases and hemicellulases are employed, to break down complex carbohydrates. Østby et al. also describe deacetylases, esterases, and lytic polysaccharide monooxygenases for the saccharification of lignocellulosic biomass (Østby et al. 2020). As it is shown in this paper, the resulting hydrolysate can be used as a medium or medium supplement for microbial cultivations without further processing steps.

### Analysis of renewable media supplements

One challenge of working with municipal green waste is the seasonality of the feedstock. At a macroscopic level, the seasonal change is obvious, with a higher proportion of hedge trimmings in spring and a significant amount of leaves in autumn. According to Boldrin and Christensen, the garden waste in Aarhus, Denmark, consisted of more than 90% of material such as grass and flowers in September, while in winter up to 45% of the garden waste consisted of woody material (Boldrin and Christensen 2010). But the composition of municipal green waste also changes on the molecular level. Over the course of the seasons, the distribution of carbohydrates, lipids, and proteins in plants varies greatly. The cell walls of young grasses make up 35% of the plant, while the cell content accounts for 65%. This ratio is inverted to 60% cell walls and 40% cell content in mature plants. As the cell walls comprise mostly lignocellulose, the overall carbohydrate content increases with maturity, while the share of proteins and lipids decreases (Sharma et al. 2011). This development also affects the composition of the media supplements derived from the biomass. The press juice obtained from mixed grass cuttings changes greatly depending on the season (see Table 1). The carbohydrate content of press juice from grass cut in spring was 53 g L^−1^, while the press juice from fall grass contained 30.5 g L^−1^ of carbohydrates. The grass stemmed from private gardens in Lohnsfeld, Germany. Although the carbohydrate content of mature plants is higher than that of young grass, the saccharides are mostly incorporated in the rigid structure of the cell walls, resulting in a lower sugar yield in the press juice from herbaceous biomass harvested in fall. The amount of press juice extracted from grass cuttings also changes depending on said factors. On average, 53% of the fresh matter can be obtained as grass press juice.

**Table 1:** Carbohydrate and protein content of press juices from different grass feedstocks

	Glucose/g L^−1^	Fructose/g L^−1^	Cellobiose/saccharose/g L^−1^	Protein (Bradford)/g L^−1^
Mixed grass cutting (spring)	19.9	29.9	3.2	0.9
Mixed grass cutting (fall)	8.5	15.0	7.0	1.0
Italian ryegrass (native)	9.0	31.1	Not quantified	1.7
Italian ryegrass (autoclaved)	7.7	22.8	Not quantified	0.5

There are already publications on the use of grass press juice as feedstock for fermentations. Schoeters et al. report 6.7 g L^−1^ of total carbon and 0.5 g L^−1^ of total nitrogen in press juice from a mixture of *Festulolium* and perennial ryegrass (Schoeters et al. 2023), while Langsdorf et al. measured 20.0 g L^−1^ total organic carbon and 1.2 g L^−1^ total nitrogen in press juice from grass clippings (Langsdorf et al. 2022).

Santamaria-Fernandez et al. reported glucose and fructose concentrations of 9–19 g L^−1^ and 7–17 g L^−1^, respectively, in mixed grass-clover press juice (Santamaria‐Fernandez et al. 2019). Hull et al. found 61.1 g L^−1^ of combined glucose, fructose, sucrose, and fructan in press juice from *Lolium perenne* (Hull et al. 2014).

Additionally to carbohydrates and proteins, grass press juice also contains various minerals and ions. The press juice of Italian ryegrass contained, among others, 2.8 g L^−1^ chloride, 6.6 g L^−1^ potassium, 997.7 mg L^−1^ sulfate, 677.6 mg L^−1^ phosphate, and 155.2 mg L^−1^ magnesium. Both Boakye-Boaten et al. and Xiu et al. give an example of the rich content of different organic acids and metabolites in *Miscanthus* press juice (Boakye-Boaten et al. 2016; Xiu et al. 2017).

For the use in cultivations, all components need to be sterilized. This also applies to media components or media supplements. However, press juice is a complex mixture containing both carbohydrates and proteins. When autoclaving amino groups together with reducing sugars, Maillard reactions are to be expected (Cardoso et al. 2023). This was shown exemplarily for one type of grass press juice. As can be seen in Table 1, the press juice of Italian ryegrass loses around 25% of carbohydrates and around 70% of proteins during an autoclaving process. Therefore, either the loss of nutrients has to be compensated for the use as a cultivation medium or another means of sterilization should be chosen, for example, sterile filtration. The latter has the benefit of conserving the full carbohydrate content of the media supplement. However, the procedure is time consuming and rather expensive, which hampers the transfer to an economical industrial process.

Both the solid residue after a pressing step and material too dry for pressing can be used as feedstock as well. Material with a high lignin content was pretreated with an organosolv procedure before enzymatic saccharification, and the press cake of herbaceous biomass was directly hydrolyzed enzymatically (see Fig. 1). Hereby, we achieved a hydrolysate containing carbohydrate concentrations of around 33 g L^−1^ from mixed wood chips as shown in Table 2. This feedstock has a cellulose content of 26%, which corresponds to a cellulose yield of around 74% after enzymatic hydrolysis (see also Varriale et al. 2022). Around 21% of the applied bio dry mass can be obtained as sugars in the hydrolysate. In the literature, the composition of the resulting hydrolysate used for cultivations is rarely given; mostly cellulose conversion rates are indicated. Bay et al. obtained glucose yields between 44.6 and 49.3% from poplar and pine wood as well as rice straw after phosphoric acid treatment and enzymatic hydrolysis (Bay et al. 2022). Cui et al. report glucose yields of up to 46.3% from paper mulberry (*Broussonetia papyrifera*) after pretreatment with deep eutectic solvents at elevated temperatures followed by saccharification (Cui et al. 2022). The hydrolysis yield of beech chips after microwave-assisted pretreatment with 60% ethanol and 1% sulfuric acid and enzymatic hydrolysis performed by Mikulski et al. is 56.8% (Mikulski and Kłosowski 2023). During hydrothermal pretreatments, sugar degradation products such as 5-(hydroxymethyl) furfural (5-HMF) and furfural as well as other phenolic compounds and acids are formed. Mikulski et al. report 5-HMF and furfural concentrations of 51.5 and 6289.7 mg L^−1^, respectively, for wheat straw, which was pretreated microwave assisted with 60% ethanol and 1% sulfuric acid, and 433.4 and 5945.9 mg L^−1^ for equally processed beech chips (Mikulski and Kłosowski 2023). In this study, the hydrolysate of mixed wood chips contained 4.1 mg L^−1^ HMF and 4.2 mg L^−1^ furfural, while the hydrolysate of a mixture of wood chips and grass only comprises 2.3 and 3.8 mg L^−1^, respectively. These degradation products inhibit fermentation processes by intercalating into biological membranes (Palmqvist and Hahn-Hägerdal 2000; Kordala et al. 2023). Different detoxification strategies such as the use of laccase, chemical and physical methods, or the in situ removal by a fermentative organism are discussed in the literature (Fillat et al. 2017; Jönsson et al. 2013; Ujor and Okonkwo 2022). In this work, a washing step between the organosolv process and the enzymatic saccharification was employed, which explains the low concentration of less than 9 mg L^−1^ of sugar degradation products left in the final hydrolysate. As the grass press cake was not pretreated hydrothermally, no sugar degradation products should be expected in the hydrolysate.

**Table 2:** Composition of enzymatic hydrolysate of different lignocellulosic feedstocks

Component	Mixed wood chips (organosolv pretreatment)	3:1 grass and wood chips (organosolv pretreatment)	Grass press cake
Glucose/g L^−1^	21.3	17.6	10.8 ± 0.1
Xylose/galactose/mannose/g L^−1^	12.3	9.2	6.7 ± 0.1
5-HMF/mg L^−1^	4.1	2.3	nd
Furfural/mg L^−1^	4.2	3.8	nd

Mixed wood chips and the wood–grass mixture were pretreated using an organosolv procedure before enzymatic hydrolysis. Organosolv pretreatment with 50% (*w*/*w*) EtOH and 10% solid loading for 15 min at 180 ℃, enzymatic hydrolysis with 0.16 g_enzyme_/g_biomass_ and 5% solid loading for 72 h at 50 ℃. Mean deviation from *n* = 2 if indicated

The pretreatment method described above, the enzymatic hydrolysis of biomass pretreated with an organosolv procedure, not only results in a hydrolysate with a 60% higher glucose yield compared to the literature, but additionally does not require an expensive detoxification step to remove sugar degradation products. Therefore, it is a suitable procedure to obtain cultivation media from municipal green waste.

In the following chapters, the general suitability of media supplements derived from municipal green waste for the use in microbial cultivations is demonstrated. For industrial processes, however, the carbohydrate concentration of the resulting media is too low to obtain satisfying product yields. One possibility to further increase the glucose concentration of the hydrolysate is the optimization of the pretreatment processes. For example, the enzyme cocktail can be modified and augmented with cellulases. Another possibility is to increase the solid loading during the enzymatic hydrolysis, which can increase the carbohydrate yield due to the additional polysaccharides present. However, a higher solid loading also increases the liquefaction time and requires more energy and possibly other equipment. Amândino et al. determined a solid loading of 11% (*w*/*v*) for the enzymatic hydrolysis of *Eucalyptus globulus* bark as an optimal trade-off between a higher sugar concentration and the hydrolysis conversion efficiency (Amândio et al. 2023). An optimization of the hydrolysis procedure is necessary to render the process more cost effective.

### Cultivation of four different microorganisms with media supplements based on municipal green waste

The suitability of media supplements derived from municipal green waste was tested using several microorganisms. The organisms were chosen diversely to cover a broad range of cultivation requirements. Both bacteria and fungi were tested, as well as aerobic and anaerobic organisms. Table 3 summarizes various publications examining the cultivation of the organisms employed here using media based on different lignocellulosic feedstocks as a comparison.

**Table 3:** Overview of publications using different lignocellulosic waste fractions as feedstock for the microbial production of various products

Substrate	Product	Organism	References
Press juice from a mixture of *Festulolium* and perennial ryegrass	1.01 g_algal biomass_ L^−1^ with 41% protein content	Co-cultivation of microalgae *Chlorella sorokiniana* and *Acutodesmus* *obliquus*	Schoeters et el. 2023
Extracted juice from mixed grass clippings	2 mg_α-humulene_ L^−1^	*Cupriavidus necator*	Langsdorf et al. 2022
Press juice of *Lolium perenne*	22.5 g_ethanol_ L^−1^, 7.89 mg_squalene_·g_dry mass_^−1^	*S. cerevisiae* YUG37-ERG1	Hull et al. 2014
Cashew apple juice	38.6 g_ethanol_ L^−1^ 0.47 g_ethanol_ g_sugar_^−1^	*S. cerevisiae*	Pacheco et al. 2010
Simulated corn stover hydrolysate	53.4 g_ethanol_ L^−1^ 0.498 g_ethanol_ g_total sugars_^−1^	Engineered *S. cerevisiae* strain	Wang et al. 2022
100% press juice from mixed grass cuttings without additional components	9.4 g_ethanol_ L^−1^ 0.61 ± 0.03 g_ethanol_ g_sugar_^−1^	*S. cerevisiae*	This work
Unsterile press juice of *Miscanthus x giganteus* (MxG) with the addition of saccharolytic enzymes	11.91 g_lactic acid_ L^−1^, 0.29 g_ethanol_ L^−1^ ethanol 0.63 to 1.38 g_acetuc acid_ L^−1^	Heterolactic co-culture of *Lactobacillus plantarum* and *Lactobacillus brevis*	Boakye- Boaten et al. 2016
Press juice of grass-clover mixtures	22 g_lactic acid_ L^−1^	*L. salivarius* BC 1001	Santamaria‐ Fernandez et al. 2019
Hydrothermally pretreated sugarcane bagasse	52.4 g_lactic acid_ L^−1^ 0.66 g_lactic acid_ g_sugar_^−1^	*B. coagulans* under non-sterile conditions	Cox et al. 2023
Chemically and enzymatically pretreated *P. australis* reed stems	28.3 g_lactic acid_ L^−1^	*B. coagulans*	2020
100% press juice from mixed grass cuttings without supplements	16.93 g_lactic acid_ L^−1^ 1.36 ± 0.04 g_lactic acid_ g_sugar_^−1^	*Lactobacillus delbrueckii* subsp. *lactis*	This work
Beech wood xylan	0.08 g_itaconate_ L^−1^	*U. maydis* MB215P_oma_xyn11A	Schroedter et al. 2016
Cellobiose	5.2 g_itaconate_ L^−1^	*U. maydis* MB215_oma_bgl1	Geiser et al. 2016
Hydrolyzed hemicellulose fraction from beech wood	0.36 g_itaconic acid_ L^−1^	*U. maydis* MB215	Klement et al. 2012
Hydrolyzed hemicellulose fraction from beech wood with added glucose	8.5 g_itaconic acid_ L^−1^	*U. maydis* MB215	Klement et al. 2012
Brewer’s spent grain, hydrothermally pretreated and enzymatically hydrolyzed	6 g_itaconic acid_ L^−1^ 0.38 g_itaconic acid_ g_sugar_^−1^	*U. maydis* MB215 ΔCyp3 P_etef_Ria1	Weiermüller et al. 2021
Mineral salt medium + 70% (*v*/*v*) press juice of mixed grass cuttings	19.18 g_itaconic acid_ L^−1^ 0.51 g_itaconic acid_ g_glucose_^−1^	*U. maydis* MB215 ΔCyp3 P_etef_Ria1	This work
Mineral salt medium with 40% (*v*/*v*) enzymatic hydrolysate of mixed wood chips after organosolv pretreatment	17.2 g_itaconic acid_ L^−1^ 0.40g_itaconic acid_ g_glucose_^−1^	*U. maydis* MB215 ΔCyp3 P_etef_Ria1	This work
Enzymatic hydrolysate of alkaline pretreated coffee silverskin	3.2 g_ABE_·L^−1^ 0.1 g_ABE_ g_sugar_^−1^	*C. acetobutylicum*	Niglio et al. 2019
Detoxified wood hydrolysate and alfalfa juice with buffering agents, vitamins and minerals	0.17 g_solvents_ g_total sugars_^−1^	*C. acetobutylicum*	Mechmech et al. 2016
Detoxified pine wood hydrolysates	5.7 g_butanol_ L^−1^	*C. acetobutylicum*	Maddox and Murray 1983
Mineral salt medium with 30% (*v*/*v*) enzymatic hydrolysate of mixed wood chips after organosolv pretreatment	15.5 g_ABE_ L^−1^ 0.31 ± 0.01 g_ABE_ g_glucose_^−1^	*C. acetobutylicum*	This work

### Ethanol production of *Saccharomyces cerevisiae* in grass press juice

In the literature are reports of various media used for the cultivation of *S. cerevisiae*. Most of the media derived from waste material are based on enzymatic hydrolysates from pretreated lignocellulosic biomass, but there are also few publications using material without prior enzymatic hydrolysis. For example, Boakye-Boaten et al. showed the suitability of *Miscanthus* grass press juice for the cultivation of *S. cerevisiae* and achieved a 75% increase in cell dry weight under anaerobic conditions compared to the growth in standard yeast medium broth. However, they did not determine the formation of ethanol using press juice (Boakye-Boaten et al. 2016). Pacheco et al. cultivated *S. cerevisiae* in cashew apple juice supplemented with media components and achieved an ethanol concentration of 38.6 g L^−1^ but obtained a yield of 0.47 g_ethanol_/g_sugar_ (Pacheco et al. 2010). Further optimization can be achieved by genetic modifications of the organism. Wang et al. engineered a *S. cerevisiae* strain to also metabolize xylose and yielded 0.498 g_ethanol_/g_total sugars_ with an ethanol concentration of 53.4 g L^−1^ in a simulated corn stover hydrolysate (Wang et al. 2022).

In this work, autoclaved grass press juice was used as medium for the anaerobic cultivation of *Saccharomyces cerevisiae* for the fermentative production of ethanol. Due to the turbidity of the autoclaved press juice, reliable measurement of the optical density (OD_600_) was not always possible. Cell growth could be observed by determining the bio-dry mass, and the metabolism could be analyzed by the substrate and product concentrations. Before a scaleup, different cultivation setups with and without additives were tested in 100 mL Schott flasks (see Table 4).

**Table 4:** Yield of ethanol for cultivations of *S. cerevisiae* in reference media and grass press juice with and without additional supplements

Cultivation conditions	g_ethanol_/g_sugar_
General yeast medium	0.68 ± 0.01
100% grass press juice without additional components	0.59
100% grass press juice with all complex media components + invertase	0.71
100% grass press juice without additional components, 0.4 L stirred tank bioreactor	0.61 ± 0.03

Mean deviation from *n* = 2 if indicated, experiments conducted in shake flasks if not stated otherwise

**Fig. 2:**
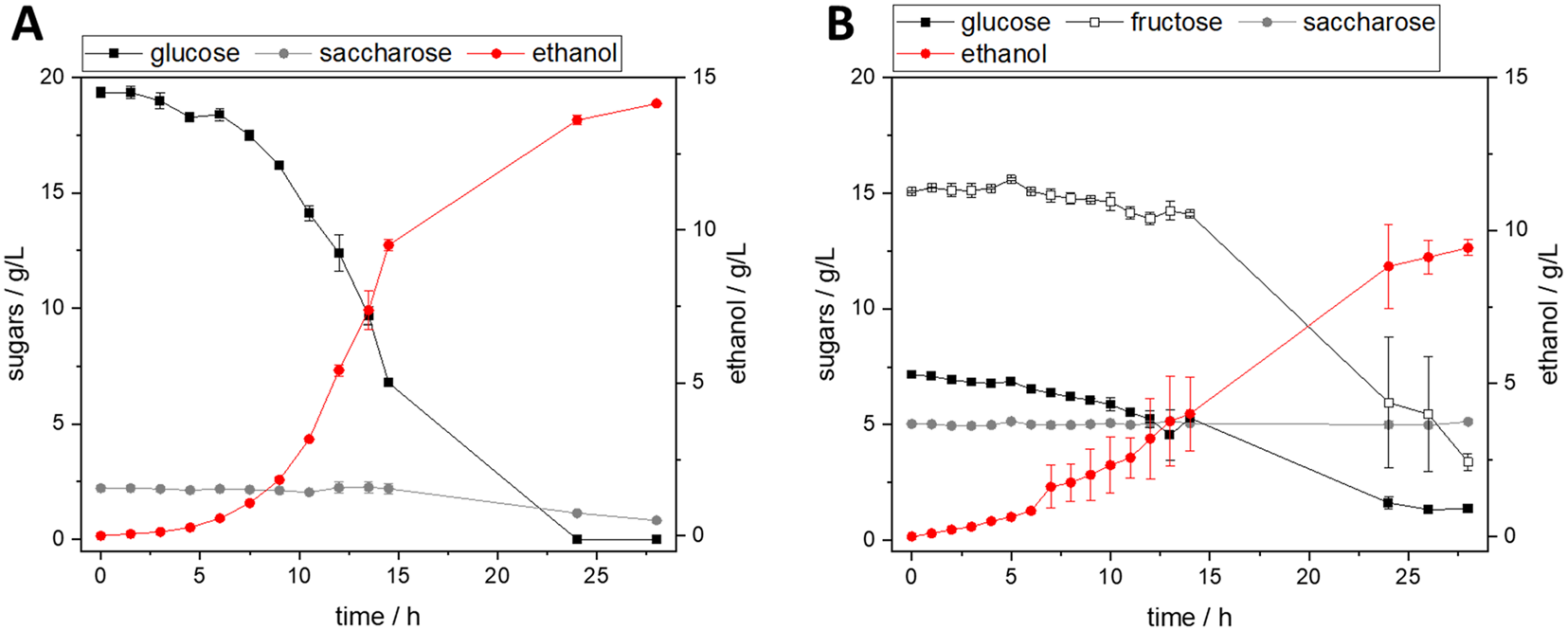
Cultivation of *S. cerevisiae* in **A** standard complex medium in Schott flasks and **B** 100% autoclaved grass press juice without any additives in a bioreactor system. *n* = 2, lines serve as a guide to the eye

*Saccharomyces cerevisiae* can both grow and produce ethanol in pure grass juice. According to the course of the ethanol and sugar concentrations (see Fig. 2B), the metabolism of the organisms is slightly slower in grass press juice, but there is no prolonged lag phase.

The press juice used for the cultivations of *S. cerevisiae* contained 5.7 g L^−1^ glucose, 13.4 g L^−1^ fructose, and 4.8 g L^−1^ saccharose. During a cultivation in pure grass press juice without any additives, both glucose and fructose were consumed almost completely (see Fig. 2B).

In contrast, saccharose was not metabolized. The addition of invertase into cultivation broths is the established basis of simultaneous saccharification and cultivation processes. Molina et al., for example, report an increase in mannitol yield of 70% produced by *Apilactobacillus kunkeei* NFICC 2128 cultivated in a mixture of apple pulp and sugar beet molasses by adding invertase to the fermentation broth (Molina et al. 2023). Therefore, the addition of invertase and media components to the grass press juice to improve the productivity and yield of the fermentation was examined in a preliminary test. While the addition of the enzyme led to the degradation of saccharose, the overall yield of the fermentation increased by only 4% (see Table 4), although components of the reference medium were supplemented as well. The marginal increase in ethanol yield does not justify the use of expensive enzymes or the addition of media components. Therefore, the scaleup in a bioreactor with a working volume of 0.4 L was performed in pure grass press juice without any additional components, which is shown in Fig. 2B. With 9.4 g L^−1^ the final ethanol concentration in grass press juice is lower than in a reference cultivation in standard medium with 14.1 g L^−1^, but with 0.61 and 0.68 g g_sugar_^−1^ the product yields of both cultivations are in the same range. The maximum theoretical yield of ethanol produced from glucose by *S. cerevisiae* is 0.51 g_ethanol_/g_glucose_ due to the production of 2 mol CO_2_. The higher yield obtained in this work traces back to the fact that the fermentation broth contained not only glucose but also fructose and other complex components which were metabolized by the microorganism. The scaleup from 50 mL in Schott flasks (see Table 4) to 0.4 L in a bioreactor system with pH control and otherwise unchanged parameters resulted in the yield increasing by 3%. This shows that pure grass press juice is a suitable medium for the cultivation of *S. cerevisiae* and can be used for the production of ethanol. For an industrial application, however, the ethanol concentration of the cultivation broth needs to be increased to render the process economically feasible.

### Lactic acid production of *Lactobacillus delbrueckii* subsp. *lactis* in grass press juice

After the successful production of ethanol using *S. cerevisiae*, the cultivation of the homofermentative *Lactobacillus delbrueckii* subsp. *lactis* using grass press juice as cultivation medium was tested for the production of lactic acid. The fermentation of grass and other lignocellulosic biomass using lactobacilli has long been known for the production of silage. Here, the acidification of foliage by an unspecific anaerobic fermentation leads to the preservation of fodder. More recent literature also shows the use of herbaceous biomass as feedstock for the cultivation of specific lactobacilli in order to produce lactic acid. Using a heterolactic co-culture of *Lactobacillus plantarum* and *Lactobacillus brevis* in unsterile *Miscanthus* press juice with the addition of saccharolytic enzymes, Boakye-Boaten et al. achieved a lactic acid concentration of 11.91 g L^−1^, together with 0.29 g L^−1^ ethanol and 0.63 to 1.38 g L^−1^ acetic acid (Boakye-Boaten et al. 2016). Santamaria-Fernandez et al. obtained lactic acid concentrations of up to 22 g L^−1^ with the organism *L. salivarius* BC 1001 in press juice of a grass-clover mixture (Santamaria‐Fernandez et al. 2019).

In this work, different parameters were examined in Schott flasks before a scaleup in a bioreactor (see Table 5). Calcium carbonate was added to both the standard complex medium and the grass press juice as a buffering agent to prevent pH shifts due to the production of lactic acid.

**Table 5:** Yield of lactic acid for cultivations of *L. delbrueckii* subsp *lactis* in reference media and grass press juice with and without additional supplements

Cultivation conditions	g_lactic acid_/g_sugar_
Complex medium + CaCO_3_	1.35
100% grass press juice + CaCO_3_	1.27
100% grass press juice with media supplements + invertase	1.85
100% grass press juice without supplements, 0.4 L stirred tank bioreactor	1.36 ± 0.04

Experiments were conducted in Schott flasks if not stated otherwise. Mean deviation from *n* = 3 if indicated

**Fig. 3:**
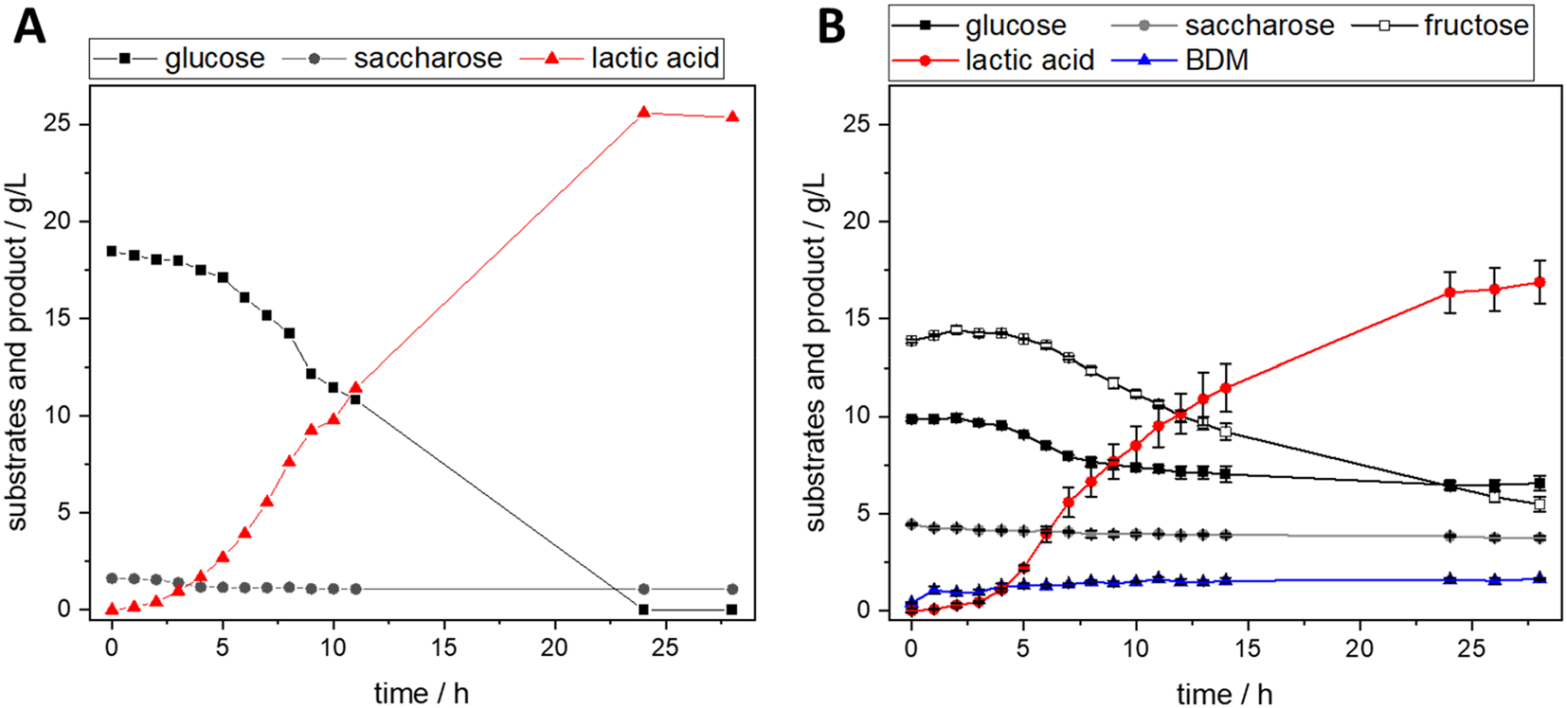
Cultivation of *L. delbrueckii* subsp. *lactis* in **A** standard complex medium with CaCO_3_ in Schott flasks and **B** 100% autoclaved grass press juice without any additives in a bioreactor system. Mean deviation from *n* = 3 if indicated, lines serve as a guide to the eye. BDM, bio dry mass

It is possible to cultivate *L. delbrueckii* in pure grass press juice and obtain lactic acid as a product. In pure press juice, the onset of the production of lactic acid is after about 5 h, whereas in the standard medium, the production starts after two hours (see Fig. 3). The addition of media components and invertase to the press juice results in the onset of lactic acid production in a comparable amount of time to in standard medium.

The yields of all experiments were above the maximum theoretical yield of 1 g_lactic acid_/g_glucose_. This is due to complex media components in the reference medium and the grass press juice which are consumed by the organism without being considered for the calculation of the yield. The addition of reference media components and invertase to convert saccharose into monosaccharides resulted in a 46% increase in yield. But as almost no degradation of saccharose could be observed, the effect is mainly attributed to the supplementation of nutrients in the form of media components. Therefore, the scaleup was performed without invertase. Moreover, due to the automatic pH regulation, calcium carbonate as a buffer could be omitted in the bioreactor cultivation. The scaleup increased the yield by 7% compared to the cultivation in Schott flasks and also resulted in a 40% increased lactic acid concentration of 16.9 g L^−1^ (see Fig. 3B). The product concentration in pure grass press juice is still lower than when cultivated in the standard medium with a final lactic acid production of 25 g L^−1^ and needs to be increased for economical industrial processes. However, this shows that grass press juice is a suitable medium for the cultivation of *L. delbrueckii* even without any supplements. The addition of media components is a possibility to increase the lactic acid yield (Table 5).

**Table 6:** Yield of itaconic acid for cultivations of *U. maydis* in media with different supplements derived from municipal green waste

Cultivation medium	g_itaconic acid_/g_glucose_
Mineral salt medium	0.28 ± 0.02
Mineral salt medium + 70% (*v*/*v*) grass press juice	0.51 ± 0.00
Mineral salt medium + 20% (*v*/*v*) hydrolysate	0.35 ± 0.00
Mineral salt medium + 30% (*v*/*v*) hydrolysate	0.42 ± 0.06
Mineral salt medium + 40% (*v*/*v*) hydrolysate	0.40 ± 0.04

Mean deviation from *n* = 2

### Itaconic acid production of *Ustilago maydis* with supplements of grass press juice and enzymatic hydrolysate

*Ustilago maydis* is a parasitic fungus that causes the so-called corn smut. Recently, the organism received more attention as it can produce itaconic acid (Geiser et al. 2014; Wierckx et al. 2020). Compared to *Aspergillus terreus*, the common producer of itaconic acid, *U. maydis* has the advantage of unicellular growth and high resistance to impurities. Therefore, *U. maydis* can be regarded as a highly robust production strain (Becker et al. 2023).

In contrast to the other organisms tested in this work, there are not many publications about the cultivation of *U. maydis* based on non-glucose carbon sources or even lignocellulosic biomass. However, as a plant pathogen, *U. maydis* is expected to be able to grow on media based on lignocellulosic biomass. Geiser et al. obtained an itaconate concentration of 0.08 g L^−1^ with an endoxylanase-producing strain MB215P_oma_xyn11A from beech wood xylan. With the strain MB215_oma_bgl1, which overexpresses a β-glucosidase, they achieved 5.2 g L^−1^ on cellobiose (Geiser et al. 2016). Klement et al. cultivated *U. maydis* MB215 on a hydrolyzed hemicellulose fraction from beech wood. With this hydrolysate being the only carbon source, the final itaconic acid concentration was 0.36 g L^−1^. The addition of glucose increased the concentration to up to 8.5 g L^−1^ (Klement et al. 2012). Using a medium based on hydrothermally pretreated and enzymatically hydrolyzed brewer’s spent grain, Weiermüller et al. achieved a yield of 0.38 g/g_sugar_, equaling an itaconic acid concentration of 6 g L^−1^ (Weiermüller et al. 2021).

**Fig. 4:**
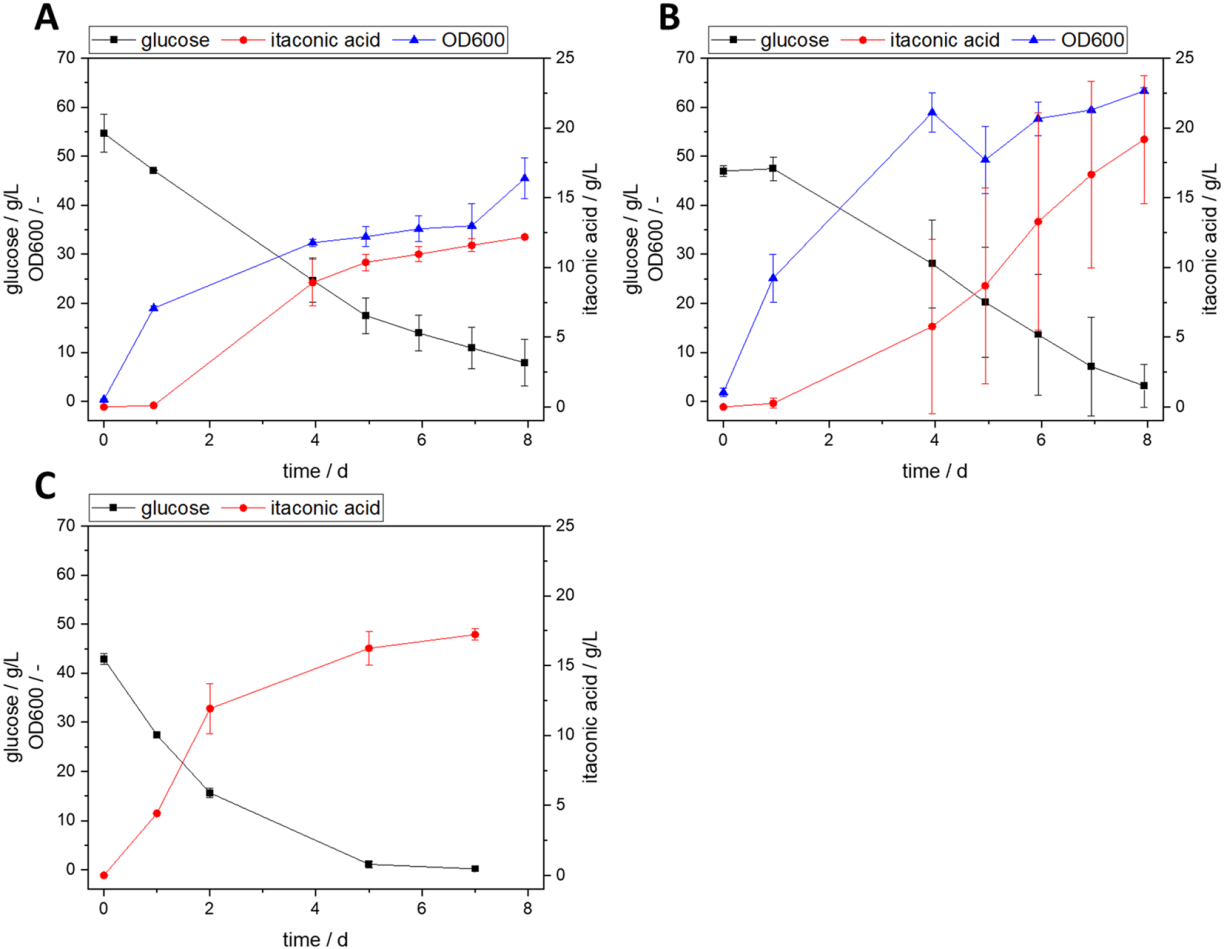
Cultivation of *U. maydis* in **A** modified Tabuchi standard medium, **B** modified Tabuchi medium supplemented with 70% (*v*/*v*) autoclaved grass press juice, and **C** modified Tabuchi medium supplemented with 40% (*v*/*v*) sterile filtered enzymatic hydrolysate of mixed wood chips pretreated with an organosolv process; *n* = 2, lines serve as a guide to the eye

Here, the *U. maydis* standard medium was supplemented with grass press juice as well as enzymatic hydrolysate of wood chips pretreated with an organosolv process in different concentrations. Due to the solubility of the buffer used for the standard medium, the amount of the supplement was limited to 70% (*v*/*v*). The glucose concentration of all experiments with media supplements was adapted to be in the range of the standard medium. *U. maydis* was successfully grown in 70% (*v*/*v*) grass press juice (see Fig. 4B).

The supplement did not affect the length of the lag phase, but the final OD reached after 8 days increased by 28%. Compared to the standard medium, the yield of itaconic acid increased by 82% to 0.51 g_itaconic acid_ g_glucose_^−1^. This considerable increase can be explained by the surplus of nutrients provided by the press juice supplementation. The press juice contains not only glucose but also fructose and complex components, which can be metabolized by *U. maydis*, but were neither considered for adjusting the sugar concentration nor for the calculation of the yield. Klement et al. demonstrated that *U. maydis* can metabolize xylose (Klement et al. 2012), and preliminary experiments indicated that *U. maydis* is able to use fructose (data not shown). So even though it is not possible to determine the precise influence on the yield, it is evident that grass press juice is a suitable medium for the cultivation of *U. maydis* and the production of itaconic acid.

Subsequently, the standard medium was supplemented with different concentrations of up to 40% of enzymatic hydrolysate of lignocellulosic biomass pretreated with an organosolv process (see Fig. 4C). Again, growth was possible and the supplementary nutrients were beneficial for the production of itaconic acid. The determination of the OD was not possible due to the formation of agglomerates when grown in the hydrolysate. However, in contrast to the cultivation in standard medium, itaconic acid is already produced within the first 24 h. Therefore, the lag phase is expected to be shorter. The yield was in the range of 0.35 to 0.42 g_itaconic acid_ g_glucose_^−1^, on average 35% higher than in the reference cultivation. Contrary to the cultivations of the previously mentioned organisms, the final product concentration of itaconic acid in cultivations with supplements of renewable media components is also higher than the concentration obtained in standard media. While the reference cultivation resulted in 12 g L^−1^, the concentration of itaconic acid in supplemented cultivations reached up to 20 g L^−1^. There is no clear trend in the itaconic acid concentration or the product yield for the experiments with 20, 30, and 40% hydrolysate supplementation (see Table 6). Preliminary tests hint that the growth of *U. maydis* in pure grass press juice as well as enzymatic hydrolysate without any additional media components is also possible (data not shown).

### Solvent production of *Clostridium acetobutylicum* with supplements of grass press juice and enzymatic hydrolysate

The organism *Clostridium acetobutylicum* is known for its so-called ABE fermentation, the fermentative production of the solvents acetone, butanol, and ethanol, typically in the ratio of 3:6:1 (Jones and Woods 1986). Especially in light of the rising demand for fuels, butanol gains in importance as a so-called biofuel. There is a long tradition of using media derived from lignocellulosic biomass for the cultivation of Clostridia (Jones and Woods 1986). Riaz et al. extensively review the use of different agro-waste feedstocks for the production of butanol using different Clostridia strains (Riaz et al. 2022). Niglio et al. focused on the use of coffee silverskin. By fermenting the enzymatic hydrolysate of this alkaline pretreated by-product from the coffee industry, they achieved an ABE concentration of 3.2 g L^−1^, corresponding to 0.1 g_ABE_/g_sugar_ (Niglio et al. 2019).

Media supplements derived from municipal green waste were successfully used to cultivate *C. acetobutylicum*. Both the addition of grass press juice and sterile filtered enzymatic hydrolysate of mixed wood chips pretreated with an organosolv process enabled the growth of the organism. For all experiments with media supplements, the final glucose concentration was adjusted to be in the same range as the reference media. Taking about 15 h, the lag phase of cultivations with green waste media supplements was prolongated by 50% compared to the standard medium. The growth phase itself lasted for 25 h, 40% longer than in the reference cultivation. The addition of media supplements also led to higher OD_600_ values of 7 and 5.5 for press juice and hydrolysate, respectively, compared to a maximum of 4.5 in the reference medium (OD_600_, data not shown). However, only marginal amounts of solvents were produced when press juice was added (see Table 7). This is likely due to a phenomenon called acid crash. Clostridia undergo a two-phased metabolism with an acid-producing acidogenesis and a solventogenesis, where the produced acids are re-assimilated and used for the formation of solvents. The acid crash describes the situation in which the metabolic switch to the second phase does not occur or the solventogenesis is terminated rapidly (Maddox et al. 2000; Kumar et al. 2013). The pH decreasing to 4.5, but especially the high concentrations of butyric acid of up to 7.7 g L^−1^ in experiments with grass press juice, supports the thesis of an insufficient metabolic switch. Two different grass press media were prepared. The standard medium components were either dissolved in 30% (*v*/*v*) press juice, hence adding additional nutrients to the standard medium recipe, or the press juice was added to readily prepare standard medium, hence diluting the standard medium components. Both the addition and the dilution lead to comparable results with low solvent and high acid yields (see Table 7). Therefore, the acid crash is not caused by a lack of nutrients from the media, but by the press juice itself. The triggers for the metabolic switch to solventogenesis as well as the reasons for the acid crash phenomenon are not finally understood yet. Although a low pH value is necessary for the onset of solventogenesis (Hüsemann and Papoutsakis 1988), an internal pH value in the cells below 5.5 hampers the enzyme activity necessary for the switch to solvent production (Gottwald and Gottschalk 1985). One possibility for the acid crash in press juice could be a reduced buffering capacity of the medium in which the pH drop cannot be compensated by the organisms through the uptake of the produced acids. As growth itself was not hampered by the press juice and butyric acid concentrations of up to 7.7 g L^−1^ were obtained, high solvent yields should be achieved by controlling the pH value during the cultivation to prevent an acid crash.

**Table 7:** Yield of ABE products for cultivations of *C. acetobutylicum* in media with different supplements derived from municipal green waste

Cultivation medium	Yield
Standard medium (see Fig. 5A)	0.34 ± 0.11 g_ABE_/g_glucose_
Reduced standard medium + 5% (*v*/*v*) grass press juice	0.18 ± 0.03 g_ABE_/g_glucose_ 0.09 ± 0.02 g_butyric acid_/g_Glu_
Standard medium + 30% (*v*/*v*) grass press juice	0.02 ± 0.01 g_ABE_/g_glucose_ 0.19 ± 0.07 g_butyric acid_/g_glucose_
Reduced standard medium + 30% (*v*/*v*) grass press juice	0.04 ± 0.01 g_ABE_/g_glucose_ 0.30 + 0.01g_butyric acid_/g_glucose_
Standard medium + 30% (*v*/*v*) hydrolysate (see Fig. 5B)	0.31 ± 0.01 g_ABE_/g_glucose_

Mean deviation from *n* = 2

**Fig. 5:**
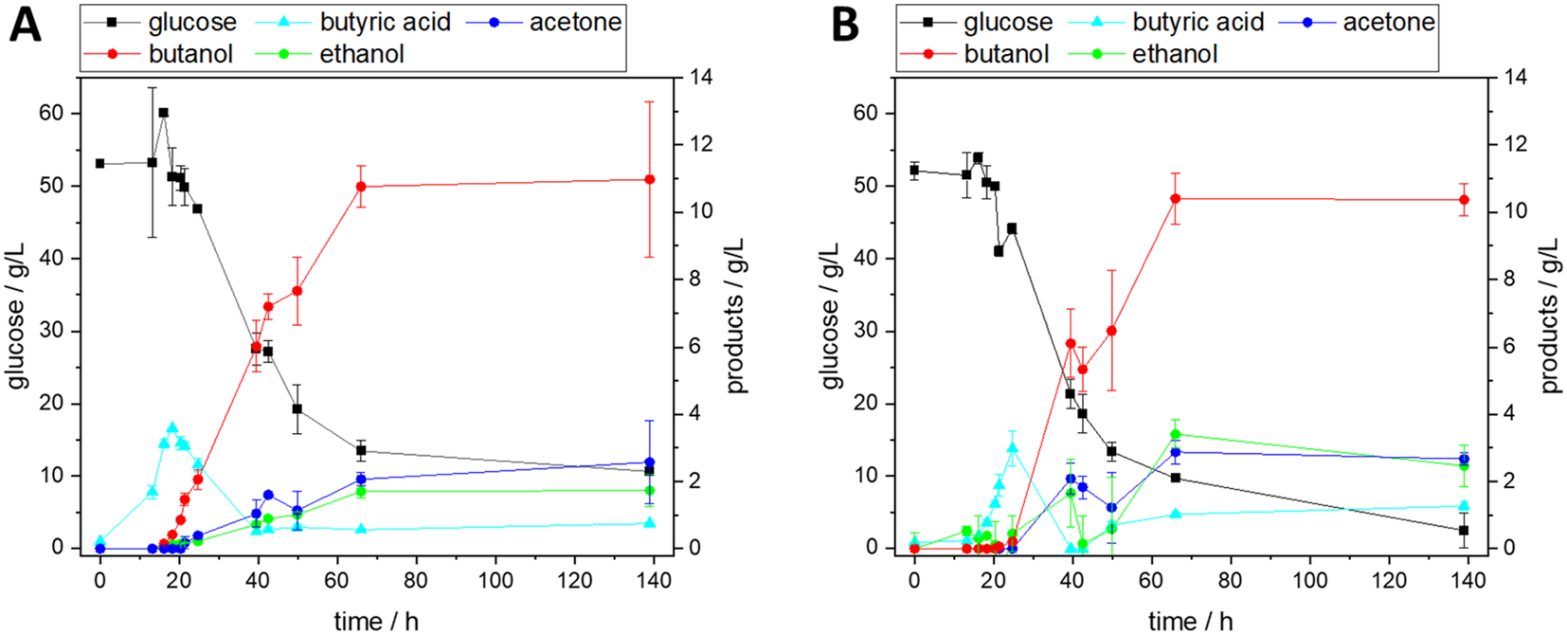
Cultivation of *C. acetobutylicum* in **A** Mp2opt standard medium and **B** standard medium supplemented with 30% (*v*/*v*) sterile filtered enzymatic hydrolysate of mixed wood chips pretreated with an organosolv process; *n* = 2, lines serve as a guide to the eye

The addition of 30% (*v*/*v*) enzymatic hydrolysate of mixed wood chips pretreated with an organosolv process had no negative influence on the growth or productivity of the organism. Apart from a slightly extended lag phase, the cultivation is nearly identical to the cultivation in the standard medium (see Fig. 5), achieving similar solvent concentrations and solvent yields (see Table 7). As the yield only refers to the consumed glucose and the hydrolysate contains also other sugars and complex components, the yield is probably slightly overestimated. Still, the enzymatic hydrolysate is a promising media supplement. While in this case only glucose was partly replaced by the supplement, the rest of the standard media components could likely be reduced or even replaced completely by the hydrolysate as well, reducing the costs for the cultivation medium.

Mechmech et al. cultivated *C. acetobutylicum* with wood hydrolysate as carbon source and alfalfa juice as nitrogen source together with buffering agents as well as vitamins and minerals and obtained a solvent yield of 0.17 g/g_total sugars_. However, the wood hydrolysate was detoxified by flocculation before its use in fermentations (Mechmech et al. 2016). Maddox et al. also report the necessity to detoxify pine wood hydrolysates. Without this pretreatment, no cultivation of *C. acetobutylicum* was possible, whereas with a combined anion and cation exchange pretreatment they achieved a butanol concentration of 5.7 g L^−1^ (Maddox and Murray 1983). In this work, however, the only detoxification procedure was a simple washing step of the hydrothermally pretreated material before enzymatic saccharification, simplifying the procedure and making it more economically feasible. Furthermore, with a butanol concentration of 10.3 g L^−1^ and an overall ABE concentration of 15.5 g L^−1^, the process presented here is also competitive.

## Conclusions

In this work, the applicability of municipal green waste as feedstock for microbial cultivations was examined. Due to the recalcitrant lignocellulose structure, pretreatment of the biomass is necessary. It should be adapted according to moisture and lignin content. It could be shown that both press juice from herbaceous biomass and enzymatic hydrolysate of high-lignin biomass pretreated with an organosolv process are suitable as a medium or medium supplement for various organisms for the production of a broad product spectrum. *S. cerevisiae* and *L. delbrueckii* were able to grow in pure grass press juice without any additives and produced 9 g L^−1^ ethanol with a yield of 0.61 g_ethanol_/g_sugar_ and 16 g L^−1^ lactic acid with a yield of 1.36 g_lactic acid_/g_sugar_, respectively. The higher than theoretically possible yield is due to additional carbohydrates in the media supplements. Thus, the yields are comparable to cultivations in standard media, although the product concentrations are lower. As the addition of further media components did not improve the cultivation process significantly, grass press juice contains enough nutrients for the growth of both organisms and the production of ethanol and lactic acid. *U. maydis* can use supplements of both grass press juice and enzymatic hydrolysate of biomass pretreated with an organosolv process as a source of nutrients for the production of itaconic acid, and the supplementation increased both product concentration and product yield from 12.0 g L^−1^ and 0.28 g_itaconic acid_/g_glucose_ to up to 20 g L^−1^ and 0.51 g_itaconic acid_/g_glucose_. The cultivation of *C. acetobutylicum* is also possible when replacing up to 30% of the standard media components with grass press juice or enzymatic hydrolysate. With the latter, product concentration and yield are similar to reference cultivations with 10.4 g L^−1^ butanol and 0.31 g_ABE_/g_glucose_. The use of grass press juice yields high butyric acid concentrations of up to 7.7 g L^−1^ and requires pH regulation for the production of butanol. The present study shows the applicability of different municipal waste fractions as a feedstock for a broad range of microorganisms. Depending on the microorganism, little to no further nutrients need to be added to the media derived from municipal green waste. This demonstrates the potential of municipal green waste as a promising feedstock for microbial fermentations.

## Materials and methods

### Raw material

Grass cuttings as well as wood chips were obtained from private gardens in Lohnsfeld and Kaiserslautern, Germany (49°33′3’’ N, 7°51′12’’ E and 49°26′5’’°N, 7°43′18°E) as a mixture of lignocellulosic biomass representative for municipal green waste. If not stated otherwise, the grass stems from meadows with a blend of grass species characteristic of typical garden and park lawns. The wood chips mainly stem from beech and pine trees.

### Production of media supplements from municipal green waste

#### Mechanical and physicochemical pretreatment

The grass was pressed using a screw press Angel Juicer 7500 (Luba GmbH, Bad Homburg, Germany), operating at 82 rpm. Biomass with a low moisture content was pretreated with an organosolv process in a high-pressure reactor BR-500 (Berghof Products + Instruments GmbH, Eningen, Germany) with a volume of 0.5 L. To prevent deformation of the reaction vessel of polytetrafluorethylene (PTFE) an overpressure of 5 bar N_2_ was applied. The reactor was heated to 180 ℃ for a holding time of 15 min using an electric heating jacket (Berghof Products + Instruments GmbH). 50% (w/w) ethanol was used as solvent with a solid:liquid ratio of 1:10. The stirring speed was 600 rpm. After cooling down, the residue was washed three times with demineralized water to remove solvent and hydrothermal degradation products.

#### Enzymatic pretreatment

Biomass pretreated with an organosolv process was enzymatically hydrolyzed using a mixture of different enzymes as described in (Varriale et al. 2022). Xylanase 2 × and Pectinase L-40 (both ASA Spezialenzyme GmbH, Wolfenbüttel, Germany), containing different xylanases and polygalacturonase, were used in a ratio of 60:40. Enzyme loading based on the protein content of the enzyme solutions was set at 0.16 g_enyzme_ g_biomass_^−1^ with a biomass loading of 5%. The biomass was autoclaved in the reaction vessel before the addition of the enzymes to prevent microbial contamination and the hydrolysis was conducted at 50 ℃ and 50 rpm for 72 h.

#### Analysis

The carbohydrate and lignin content of the biomass was determined according to the protocol of the National Renewable Energy Laboratory (Sluiter et al. 2012). Quantitative analysis of sugar monomers and fermentation products was conducted using an HPLC system [ESA Inc. 542 autosampler (Chelmsford, Massachusetts, USA), Azura pump P 6.1 L (Knauer GmbH, Berlin, Germany)] with a refractive index detector. The Bio-Rad Aminex HPX-87H column (300 × 7.8 mm, Hercules, California, USA) had a temperature of 80 ℃. 2.5 mM H_2_SO_4_ with a flow rate of 0.6 mL min^−1^ was used as the mobile phase. The separation of xylose, galactose, and mannose was not possible with this system. As it is the most common sugar of the three, the respective peaks were identified as xylose, but presumably it is a mixture of the pentoses.

#### Organisms

*Saccharomyces cerevisiae* (DSM 3799), *Lactobacillus delbrueckii* subsp. *lactis* (DSM 20072), and *Clostridium acetobutylicum* (DSM 792) were obtained from DSMZ (Deutsche Sammlung von Mikroorganismen und Zellkulturen, Braunschweig, Germany). *Ustilago maydis* MB215 ΔCyp3 P_etef_Ria1 was kindly provided by Prof. Lars Blank from RWTH Aachen. All organisms were stored as cryo cultures at – 80 ℃ in the respective culture medium with 40% glycerol.

*Saccharomyces cerevisiae* was cultivated anaerobically in 100 mL Schott flasks with butyl septa at 32 ℃ and 120 rpm in 50 mL General Yeast Medium containing 5 g L^−1^ casein peptone, 3 g L^−1^ yeast extract, 3 g L^−1^ malt extract, and 20 g L^−1^ glucose at pH 6.5.

*Lactobacillus delbrueckii* subsp. *lactis* was cultivated anaerobically in 100 mL Schott flasks with butyl septa at 45 ℃ and 120 rpm in 50 mL MRS medium as recommended by the DSMZ containing 10 g L^−1^ tryptically digested casein peptone, 10 g L^−1^ meat extract, 5 g L^−1^ yeast extract, 2 g L^−1^ potassium phosphate, 5 g L^−1^ sodium acetate, 2 g L^−1^ tri-ammonium citrate, 0.2 g L^−1^ magnesium sulfate heptahydrate, 0.05 g L^−1^ manganese sulfate monohydrate, 1 g L^−1^ polysorbate 80, and 20 g L^−1^ glucose at pH 7.0.

*Ustilago maydis* was cultivated aerobically in 500 mL shake flasks with baffles at 30 ℃ and 120 rpm. Pre-cultures were grown in 150 mL YEPG medium containing 10 g L^−1^ yeast extract, 20 g L^−1^ casein peptone, and 20 g L^−1^ glucose. They were used to inoculate 200 mL of modified Tabuchi medium to OD_600_ 0.3. The medium contains 19.5 g L^−1^ MES buffer, 50 g L^−1^ glucose, 0.8 g L^−1^ ammonium chloride, 0.5 g L^−1^ monopotassium phosphate, 0.2 g L^−1^ magnesium sulfate heptahydrate, 0.1 g L^−1^ iron sulfate heptahydrate, vitamins, and trace elements at pH 6.5 (Geiser et al. 2014). When supplementing the medium with grass press juice or enzymatic hydrolysate, the final glucose concentration of the medium was adjusted to the standard concentration of 50 g L^−1^.

*Clostridium acetobutylicum* was cultivated anaerobically in 250 mL serum flasks with butyl septa at 37 ℃ and 50 rpm. Pre-cultures were grown in 100 mL modified PY + X medium containing 5 g L^−1^ tryptone, 5 g L^−1^ peptone, 10 g L^−1^ yeast extract, 5 g L^−1^ glucose, and 40 mL L^−1^ salt solution, consisting of 0.25 g L^−1^ calcium chloride dihydrate, 0.5 g L^−1^ magnesium sulfate heptahydrate, 1 g L^−1^ potassium dihydrogen phosphate, 1 g L^−1^ dipotassium phosphate, 10 g L^−1^ sodium bicarbonate, and 2 g L^−1^ sodium chloride. Experimental cultivations were inoculated with 10% (*v*/*v*) and grown in 150 mL MP2opt medium according to Engel (2019) containing 2.2 g L^−1^ ammonium acetate, 0.5 g L^−1^ potassium dihydrogen phosphate, 0.5 g L^−1^ dipotassium phosphate, 0.2 g L^−1^ magnesium sulfate heptahydrate, 0.015 g L^−1^ iron sulfate heptahydrate, 0.01 g·L^−1^ manganese sulfate monohydrate, and 1 mL L^−1^ vitamin solution, consisting of 2 mg L^−1^ 4-aminobenzoic acid, 2 mg L^−1^ thiamine HCl, and 0.01 mg L^−1^ d-biotin. The final glucose concentration of the medium was adjusted to 45 g L^−1^ for all experiments when grass press juice or enzymatic hydrolysate was added.

The scaleup of selected cultivations was conducted in a Biostat Q plus reactor system (Sartorius AG, Göttingen, Germany) with a total volume of 0.4 L with integrated temperature probe and pH probe (Easy Ferm Bio HB K8 120, Hamilton, Bonaduz, Switzerland).

## Data Availability

All data supporting the findings of this study are available in the article, supporting information, or upon request from the corresponding author.

## Abbreviations

5-HMF: 5-(Hydroxymethyl)furfural
BDM: Bio dry mass
DM: Dry matter
MxG: *Miscan* *thus* *x giganteus*
OD_600_: Optical density at 600 nm
SDG: Sustainability development goals
